# ‘To more than I can be’: A phenomenological meta-ethnography of singing groups for people with chronic obstructive pulmonary disease

**DOI:** 10.1177/1363459320978520

**Published:** 2020-12-15

**Authors:** Heather Yoeli, Jane Macnaughton

**Affiliations:** Durham University, UK

**Keywords:** arts in health, breathlessness, COPD, meta-ethnography, phenomenology

## Abstract

Anecdotal experience and qualitative accounts suggest that singing groups, classes or choirs specifically for people with COPD (henceforth referred to as COPD-SGs) are effective in improving health. However, this is not reflected in the quantitative evidence. This meta-ethnography deployed phenomenological methods to explore this discrepancy. Analysis identified the phenomena of *being together*, *being uplifted* and *being involved* as central benefits of COPD-SGs. When viewed through the phenomenological lens of *body-social* as distinct from *body-subject* and *body-object*, findings demonstrated that the qualitative effectiveness of COPD-SGs is greatest on a collective basis. Qualitative research into the effectiveness of COPD-SGs offers more favourable results because phenomenological approaches can identify collective benefits that quantitative methods cannot. COPD-SGs should seek to maximise these collective benefits by rediscovering their cultural and artistic heritage within the national and global Arts in Health (AiH) movement, which has long emphasised the radical creative and healing power of group activity.

## Introduction

You raise me up, so I can stand on mountains

You raise me up, to walk on stormy seas

I am strong, when I am on your shoulders

You raise me up, to more than I can be^1^

Across cultures and societies worldwide, music expresses what it means and how it feels to be human. Everyone with functioning vocal chords can create and modify rhythm, pitch and tone and so the human voice is an instrument which even the most breathless of individuals can use together.

Chronic obstructive pulmonary disease (COPD) is a progressive lung condition leading to life-limiting breathlessness and disability and to complex health and care needs (WHO, 2019). It is an illness largely associated with disadvantage, marginalisation and stigma and people living with COPD report high levels of anxiety and isolation (Williams and Carel, 2018), and thereby feelings of invisibility, diminished self-confidence and disempowerment (Carel et al., 2015; Macnaughton, 2020). Affected individuals who participate in singing classes, groups and choirs for people with COPD (henceforth referred to as COPD-SGs) frequently report meaningful and lasting wellbeing and health benefits (Goldenberg, 2018). However, these benefits are rarely reflected in quantitative indices of lung function (Lewis et al., 2016). As a result, evidence bases generated from systematic reviews tend to be weak (McNamara et al., 2017). This research questions this apparent mismatch between clinical signs and patient experience and so to understand these qualitative benefits that COPD-SG participants experience.

Qualitative research into lung disease increasingly employs phenomenological methods as a means of engaging not only with the physiological or psychological aspects of life with chronic illness but also with how breathlessness affects more fundamental questions of human embodiment and interrelatedness (Macnaughton and Carel, 2016). This meta-ethnography (France et al., 2016; Noblit and Hare, 1988) uses phenomenology to explore the qualitative research informing the lived experiences of COPD-SGs to both individuals and groups. By contextualising our findings within the cultural and ideological heritage of the music in health (MiH) and arts in health (AiH) movements, we argue that COPD-SGs are qualitatively effective because they facilitate a communal or collective experience of *being* and the *body-social* that enables individuals to transcend the isolation and disempowerment of their illness. Our assertion that the qualitative benefits of COPD-SGs operate primarily on a collective or social level will be valuable to clinicians and musicians alike in informing both the commissioning and delivery of COPD-SG activities.

## Background

### Collective and individual standpoints

Within the artistic community, the effectiveness of improving collective wellbeing and health by using music to strengthen and empower communities has long been acknowledged (Stacy et al., 2002). In its earliest form, the AiH movement, including MiH, originated largely from a disparate selection of socially-engaged and often dissident or so-called ‘Outsider’ artists seeking to improve the wellbeing of under-resourced and intersectionally-disadvantaged groups across the world by using their creative practice in advocacy or activism to promote critical consciousness and social justice (Raw et al., 2012). At its inception in the late 1990s and early 2000s, the concepts of social prescribing (Brandling and House, 2009) and arts on prescription (Bungay and Clift, 2010) sought to develop mechanisms through which primary care practitioners could refer patients to AiH, MiH and other community activities, aiming both to benefit the wellbeing of individuals patients and to promote cultural capital and social cohesion. Accordingly, the five broadest literature reviews of MiH have found that activities deliver their greatest benefits on a collective level, and are usually more effective in strengthening and empowering communities than in treating individual symptoms of illness (Clark and Harding, 2012; Clements-Cortés, 2015; Clift et al., 2011; Daykin et al., 2018; Reagon et al., 2016). Nevertheless, the increasing behaviourism and individualisation of primary care, both globally and in the UK, has led public health policy and practice to re-conceptualise social prescribing as units of ‘treatment’ to address specified symptoms (DoH, 2004). As a result, AiH and MiH groups have been incentivised to re-conceptualise their artistic practices in therapeutic terms in order to receive statutory funding for their work, and in so doing to collaborate more closely with clinicians (Raw et al., 2012). It is out of this collaboration and innovation that the various models of COPD-SG provision have emerged, together with the research and evaluation that continues to inform their development (Lewis et al., 2016). Social prescribing, and its accompanying debates around autonomy, governance and the aims and value of the arts, continues to underpin much of the current COPD-SG and wider AiH practice (Yoeli et al., 2021).

Despite the increasing levels of collaboration, partnership and innovation within this field, COPD-SGs (and MiH and AiH more generally) must nevertheless be distinguished from music therapy and from the arts psychotherapies and other arts-based medical treatments in general. The arts psychotherapies, like most forms of medical treatment, are individually task-orientated in its aim to treat symptoms, improve wellbeing, promote coping and target behaviour change. AiH, MiH and COPD-SGs, by contrast, are collaborative and process-driven, aiming foremost to generate a genuine and meaningful artistic experience and only as a by-product of that artistic experience to elicit wellbeing benefits. Whereas the arts psychotherapies are delivered by allied health practitioners working within the boundaries and structures of their profession, AiH, MiH and COPD-SG activities are facilitated by practising artists whose creative expertise and freedom from healthcare conventions enables mutuality, collaboration and co-creation with group members.

### Quantitative and qualitative findings

Four recently-published literature reviews examining the evidence informing COPD-SGs have focused specifically upon COPD-SG outcomes for individuals. Each of these (Gick and Nicol, 2015; Goldenberg, 2018; Lewis et al., 2016; McNamara et al., 2017) found that COPD-SGs generally did little to improve the lung function of participants or to improve general mobility, exercise tolerance or walking distance.

Evidence of the effect of COPD-SGs on mental health and health-related quality of life varied in accordance with the measures used. In relation to all benefits of COPD-SGs, the qualitative data also offered stronger evidence than the quantitative. The experience of chronic breathlessness affects cognition, mood, emotion, coping and lifestyle in more complex and nuanced ways than structured clinical questionnaires can capture (Williams and Carel, 2018).

None of these four reviews addressed the problem from which this meta-ethnography began: the disparity between the relatively equivocal quantitative data and the strong, mainly anecdotal qualitative benefits. The empiricism upon which quantitative research is based relies upon sensory observations, and much of the value of singing, music and the arts rests upon its subjective capacity to influence humans aesthetically, emotionally and spiritually (Clift et al., 2011).

### Phenomenology and beyond: Being a person with breathlessness; being the body-subject, body-object and body-social

Phenomenology, as propounded by Husserl (1900) addresses the limitations of empiricism because, both ontologically and methodologically, it accords reality and credibility and assigns structure to all aspects of consciousness and lived experience (Merleau-Ponty, 1945). Heidegger (1927) advances a more complex and more nuanced or ambivalent position, in which phenomenology is both an internal representation as well as a direct encounter between being or existence, and reality. Heidegger emphasises the importance of ontology, or being, as a way of adding philosophical rigour to Husserl’s foundational assumption that phenomena and reality can be scientifically relied upon to correspond with one another, blurring the distinction between mind and reality. In one sense, Merleau-Ponty (1945) stands in direct lineage to Husserl in the way that he introduces Husserl’s phenomenologies of sensation and perception into broader existentialist and scientific discourses on human cognition and the self. In developing existential perspectives on the body, however, Merleau-Ponty develops a phenomenology that relies upon Heidegger’s principle that existing and being are not singular or single-faceted entities.

When approached phenomenologically, breathlessness generally appears the most distressing and disabling feature of chronic lung disease (Williams and Carel, 2018). Furthermore, COPD patients often feel more breathless and more disabled by their breathlessness than measures of their lung function might predict, something that is generally attributed to the difficulties with interoceptive awareness that often accompany chronic illness which are often compounded by accompanying conditions such as depression and anxiety (Macnaughton, 2020). People living with breathlessness experience high levels of anxiety and isolation attributable both to their sensations of struggling for breath and to the social disadvantage associated with their illness (Williams and Carel, 2018).

Informing how phenomenology might approach the study of medical (Leder, 1984) and charismatic healing (Csordas, 1993), the work of Merleau-Ponty has increasingly been used to foreground the value of the *body-subject* or lived body of first-person experience as distinct yet inseparable from the *body-object* of the medical gaze. For people living with long-term medical conditions, however, the experience of bodily impairment and reliance upon medical care can confound this dichotomy, compelling the individuals in question to engage with their bodies simultaneously as both a passive *object* of scrutiny and care and as an autonomous *subject* in possession of consciousness and agency (Edwards, 1998).

The concept of the body as both individual and a communal, collective or social entity – the body-social – has its roots both beyond and within phenomenology. The concept has assisted anthropologists to challenge or reject the existence or *being* of an individual self or body, regarding instead a person as part of or inseparable from the family, community or society, in relation to a distinctive culture, with existence formed within the interaction between self and other (Latour, 2004; Scheper-Hughes, 1994; Scheper-Hughes and Lock, 1987). By introducing phenomena of *Mitsein* (*being with others*) and *In-der-Welt-sein* (*being in the world*) as essential components of *Dasein*, Heidegger (1927) acknowledges this, describing the nature and meaning of *being* as shaped by human relatedness to and articulation with the world. The complexity of communal, collective or social ways of *being* a body are further developed by Sartre (1943) in his analysis of what Merleau-Ponty (1968) subsequently conceptualises as *intercorporeity*, or of individual human perceptions of their own existence as being interdependent upon those of others.

Although identifying Heidegger as the inspiration for much of Merleau-Ponty’s perspectives on the body, we acknowledge the complex, nuanced and often divergent relationship between their phenomenological methods. By asserting the importance of a *body-social* as a companion to the *body-subject* and *body-object*, we lay no claim to ontological innovation. Instead, we argue that the more conventional *body-subject*/*body-object* dichotomy of phenomenological health research (Csordas, 1993; Leder, 1984) is insufficient to capture the communal, collective or social aspects of bodily experience conceptualised within *Mitsein* (Heidegger, 1927) and *intercorporeity* (Merleau-Ponty, 1968).

The following outline of the meta-ethnographic methodology and methods employed by this study explains how the approach of Merleau-Ponty and ontological questions around *being* and the *body* came to frame this study.

## Methods

### Meta-ethnography

Meta-ethnography, as a research method first proposed by Noblit and Hare (1983), is a technique for reviewing, synthesising and appraising qualitative studies which aims to remain unconstrained by the interpretations of primary authors and in so doing to identify over-arching attitudes, theory or phenomena. In contrast with other forms of literature review, meta-ethnography enables a focus on process as well as content, placing context-dependent findings within broader and more up-to-date clinical and social discourses (France et al., 2016). It is thereby ideally suited to the study of social prescribing projects and similar social initiatives seeking specific health improvements for particular populations (Pollard et al., 2020). Meta-ethnography may therefore identify findings and generate conclusions which may differ from those of the individual authors of the original sources (Lamb et al., 2012; Smithson et al., 2012). This study selected meta-ethnography as a research method, then, as an attempt to understand and to contextualise in the widest possible terms why, irrespective of equivocal previously-reviewed quantitative findings, COPD-SGs appear to produce such qualitative benefits to individuals and to groups.

### Literature searches

Traditionally, meta-ethnographies have tended to review and to synthesise only research undertaken within an interpretatively qualitative paradigm. However, a number of recent studies (Hardman et al., 2020; Sandelowski et al., 2012) have challenged this convention, arguing that the increasingly abductive logic of the meta-ethnographic process enables also the potential inclusion of all forms of qualitative and mixed-methods study. By adhering to this broader understanding of meta-ethnography’s scope and remit, this study was able to include the qualitative research undertaken within positivist and realist evaluations (Lord et al., 2012; Skingley et al., 2013) designed to generate the most widely-accepted evidence to inform COPD-SG outcomes.

In their conventional use within health research, meta-ethnographies have sought to assert their methodological legitimacy by adhering to systematic procedures for identifying relevant literature (Campbell et al., 2003; Malpass et al., 2009). Several recent methodologists, however, have challenged the philosophical incongruity of such potentially inflexible structures, arguing instead that meta-ethnographies should adopt broader and more iterative search criteria (Andersen et al., 2019; Thorne, 2017). This meta-ethnography sought to forge a middle path between these two positions, employing a systematically rigorous protocol to identify the most widely accepted publications on the topic (e.g. Lord et al., 2012; McNaughton et al., 2016; Skingley et al., 2013, 2018) but also the flexibility to include both some of the ‘grey literature’ (e.g. NZMOH, 2016; SfBB, 2016a, 2016b, 2016c) that arose alongside academic publications and those studies that conventional protocols would consider less empirically rigorous (e.g. Lewis and Thomas, 2018).

**Figure 1.**
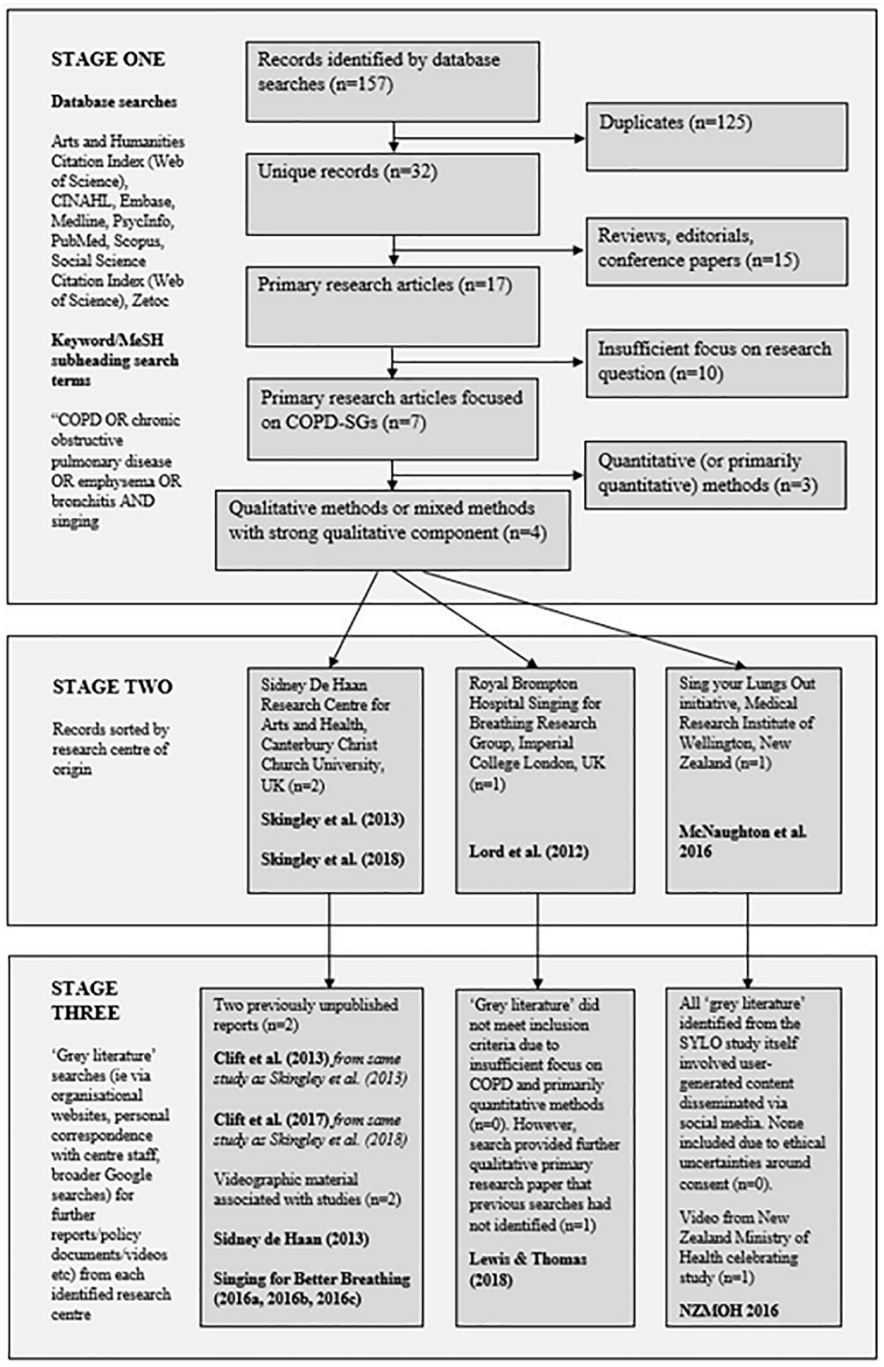
Search protocol for meta-ethnography.

Figure 1 describes the process by which we identified literature for inclusion in this meta-ethnography.

#### Stage 1

Initially, we undertook a number of indicative and experimental searches using a range of databases and search strategies. Proceeding in the systematic manner advocated by the earliest proponents of health meta-ethnography (Campbell et al., 2003; Malpass et al., 2009), we used these to identify the most relevant databases (CINAHL, Embase, Medline, PsycInfo, PubMed, Scopus and the Arts and Humanities and Social Sciences Citation Indices of Web of Science) and the most specific search terms (COPD OR chronic obstructive pulmonary disease OR emphysema OR bronchitis AND singing – inputted as MeSH subheadings and/or fulltext keywords, depending upon database capacity). As Figure 1 illustrates, this search protocol yielded 32 unique records, of which 17 were primary research studies, each of which were read in detail. Given the theoretical process employed to guide our meta-ethnography (see following sub-section), we required for inclusion only those primary studies which described or discussed their COPD related findings in sufficient depth to enable rigorous phenomenological analysis; as a result, 10 studies were excluded for their insufficient focus on COPD-SGs and three were excluded for their paucity of qualitative detail. Four studies, then, were included at this stage (Lord et al., 2012; McNaughton et al., 2016; Skingley et al., 2013, 2018)^2^.

#### Stage 2

The four hitherto included studies were sorted by research centre provenance. Two studies were identified as originating from the Sidney de Haan Research Centre (SdHRC) at Canterbury Christ Church University, UK (Skingley et al., 2013, 2018), one from the Singing for Breathing Research Group at the Royal Brompton Hospital and Imperial College London, UK (Lord et al., 2012), and one from the Sing Your Lungs Out (SYLO) choir studied by the Medical Research Institute of New Zealand (MRINZ) in Wellington (McNaughton et al., 2016). We used these three research centres as the starting points for the broader and more flexible ‘grey literature’ searches advocated by more recent meta-ethnographies (Andersen et al., 2019; Thorne, 2017). We became aware at this stage that our phenomenologically-orientated theoretical standpoint had created a search strategy which had inadvertently and unintentionally led to an Anglocentric bias with the literature that could not fully be mitigated by the high number of Maori participants in McNaughton et al. (2016). In our discussion the limitations of this study, we reflect upon this further.

#### Stage 3

We began our search for relevant ‘grey literature’ from the websites of the SdHRC, SfBRG and MRINZ, exploring material provided and suggested by individuals associated with each research centre, and undertaking Google searches to investigate further questions arising. From the SdHRC, this led to the inclusion of the two evaluation reports (Clift et al., 2013, 2017) upon which the two publications of Skingley et al. (2013, 2018) were based, as well as the videographic material produced by the SdHRC to accompany these reports (SdHRC, 2013; SfBB, 2016a, 2016b, 2016c). From the SfBRG, this led to no ‘grey literature’ but instead one further academic research study (Lewis and Thomas, 2018) which adhered to all of our Stage 1 inclusion criteria yet did not appear to have been indexed by our previously-used search engines. From the MRINZ, this identified a YouTube clip of SYLO members receiving volunteering awards (NZMOH, 2016) as well as several other pieces of social media material. After careful deliberation, we decided to include within this meta-ethnography only the former on the basis that, as a video published by the New Zealand Ministry of Health, its posting online was likely to have adhered to ethical procedures. With regard to the other social media material, we could find no means to establish whether or how participant consent may have been sought.

### Phenomenological analysis

Within this study, data analysis followed broadly the framework for health meta-ethnography of France et al. (2016). Firstly, all studies meeting the inclusion criteria were read closely, and the main findings from each identified. At its inception, meta-ethnographies of health research tended to adhere to Grounded Theory-based principles of straightforwardly inductive analysis (Campbell et al., 2003), seeking to use meta-ethnographic findings to generate knowledge without reference to existing theoretical constructs. As the methodology has broadened and diversified, however, meta-ethnography has become increasingly open to range of approaches to interpretative and standpoint analysis (Andersen et al., 2019; Hardman et al., 2020). We selected a phenomenological form of analysis on the basis of what this paper has previously described as phenomenology’s ability to transcend the limits of empiricism (Carel, 2018). We posited that, by transcending the limits of empiricism, this study could identify effects of COPD-SGs which might explain the mismatch between quantitative and qualitative evidence of their effectiveness. We adhered to Merleau-Ponty’s (1945) existential and embodied approach to phenomenological analysis on the basis of our emerging finding that concepts of *being*, and of how COPD-SGs affected individual and collective understandings of their bodies, featured predominantly throughout the literature this meta-ethnography reviewed.

Methodologically, Merleau-Ponty (1945) follows the philosophical tradition of Husserl (1900) in the way that he affirms and endorses Husserl’s concepts of sensation, consciousness and their relative roles in understanding the phenomena of lived human experience. Phenomenological methods within this tradition advocate *phenomenological reduction*: data analysis by the abstraction of experiences from experiencers, thereby identifying phenomena through establishing intersubjectivities that are purely transcendental in nature. As such, having identified the key findings from each study, this meta-ethnography sought to analyse emerging themes in isolation from the interpretations or conclusions placed upon it by their original authors. Themes within the data were then established by focusing upon their *intersubjectivity*, or upon identifying those phenomena that the researchers identified or interpreted as having been experienced within two of more of the COPD-SGs studied.

### Reflexive positioning

Phenomenological research acknowledges that both the researcher and the researched share a common experience of being human (Creswell and Poth, 2016). As such, research findings may become not only intersubjective (experiences shared by a group of participants) but also subjective (experiences given prominence on the basis of the researchers’ backgrounds and interests). Meta-ethnography, within which the researched becomes a series of written texts open to a range of potential interpretations, places a particular responsibility upon phenomenological researchers to reflect upon their positioning and to discuss potential sources of bias.

**Figure 2.**
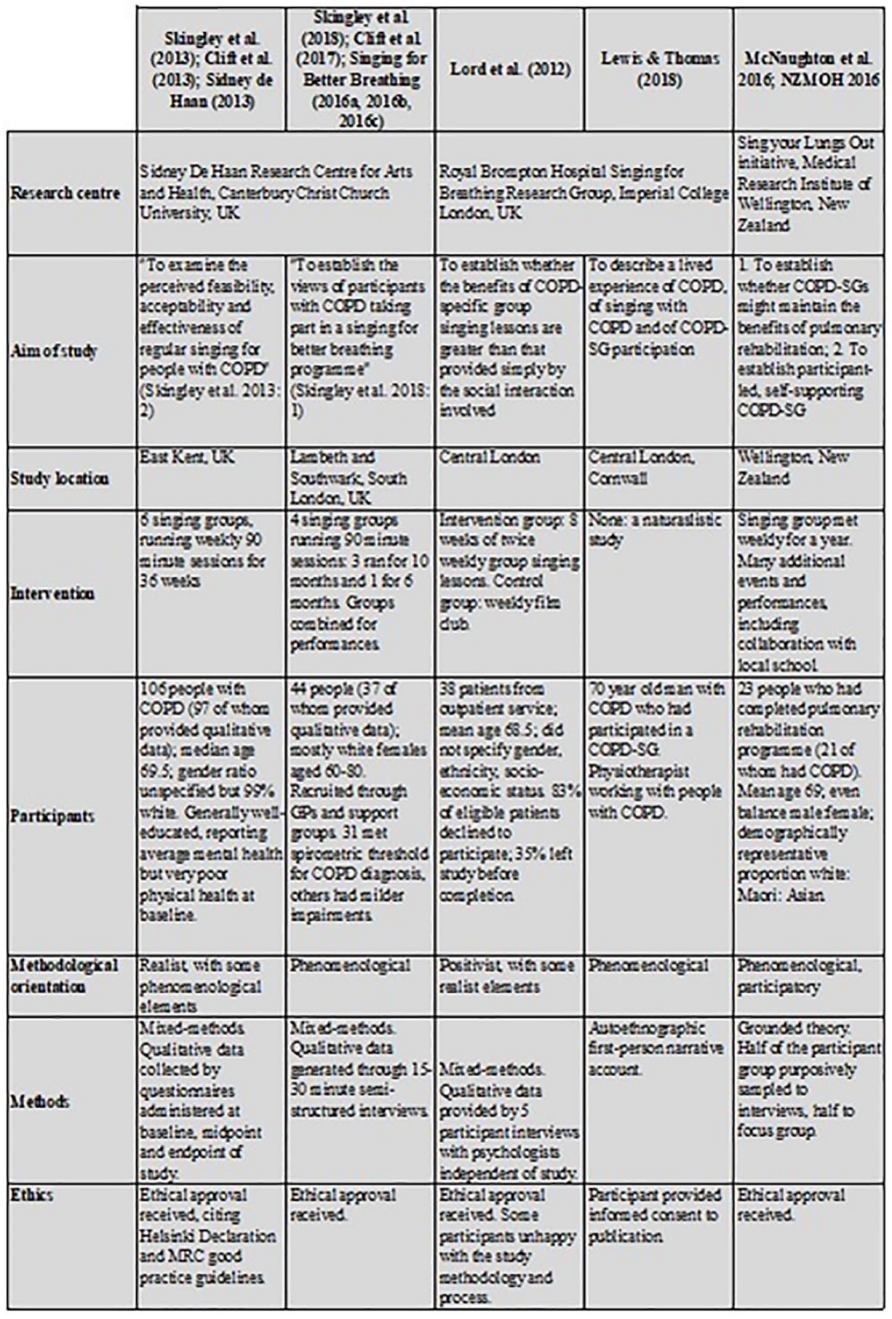
Summary of included studies.

Within the UK, MiH research is often undertaken by individuals who are either accomplished musicians who have experienced for themselves the benefits of music to their lives, or by healthcare practitioners with a clinical interest in the patient group being studied. Within our research, the lead author was neither, something which freed her from a felt need to ‘prove’ why COPD-SGs might ‘work’. This meta-ethnography was undertaken as part of a much larger study investigating the embodiment of breathlessness and related symptoms, seeking ultimately to understand how people living with COPD and similar conditions experience *being* in their bodies (Figure 2).

## Findings

### Incorporating the body-social

Through according ontological parity to the *body-subject*, *body-object* and *body-social*, it became apparent from the early stages of data analysis that the main impact of singing classes, groups and choirs for people with COPD (henceforth referred to as COPD-SGs) occurred on a communal or collective group-based basis. Analysis identified the phenomena of *togetherness, uplifted-ness* and *involvement* within the data. Each of these were framed within the concept of *being* to emphasise their intersubjective existence and reality within our phenomenological paradigm.

### Being together

Participants from all studies (Lewis and Thomas, 2018; Lord et al., 2012; McNaughton et al., 2016; Skingley et al., 2013, 2018) emphasised the value of *togetherness* in enabling them to transcend what they described as the grim experience of daily breathlessness. As individuals, participants valued their connection and commonality with one another. As groups, they preferred to identify as choirs rather than as support groups or as patient socials. Singing together, participants explained, unites people across social, cultural and ethnic backgrounds in ways that simply suffering from a common illness cannot (McNaughton et al., 2016; Skingley et al., 2018).

Participants attributed their *being together*, something particularly apparent when COPD-SGs were preparing for performances (SfBB, 2016b, 2016c), as challenging the loneliness and isolation which had typified their prior experiences of COPD (Skingley et al., 2018). Within some studies (Lewis and Thomas, 2018; McNaughton et al., 2016) belonging to their COPD-SG transcended not only a sense of personal or physical isolation, but also of their disconnectedness from their wider community. Although the SfBRG model of delivering singing *classes* did not involve performances, both studies found that participants nevertheless initiated and created their own social and musical opportunities beyond the group through informal excursions to ‘open mic’ nights in local pubs (Lewis and Thomas, 2018; Lord et al., 2012).

Within the Sing Your Lungs Out (SYLO) choir studied by the Medical Research Institute of New Zealand (MRINZ) in Wellington and the studies from the Sidney de Haan Research Centre (SdHRC) at Canterbury Christ Church University, UK (McNaughton et al., 2016; Skingley et al., 2013, 2018), it was through *being together* that participants gave and received emotional and practical support. Within the SYLO study (McNaughton et al., 2016), COPD-SG participants worked together to produce group newsletters, organised transport and catering rotas, and arranged birthday celebrations. McNaughton et al. attributed this to the emphasis within Maori culture on mutuality of support. However, although the white British culture from which most SdHRC participants originated embodies fewer such social obligations, the warm and detailed descriptions of the *togetherness* developed between group members appeared equally intense, rewarding and supportive as those in the SYLO study, though perhaps less structured. Whereas the cultural influences with the SYLO study may have engendered formalised support mechanisms, the parallels between the SdHRC and SYLO study findings suggest that this mutual supportiveness derived instead from the sense of meaning, purpose and social inclusion that came from *being together*.

### Being uplifted

All studies found that participants enjoyed singing together (Lewis and Thomas, 2018; Lord et al., 2012; McNaughton et al., 2016; Skingley et al., 2013, 2018). Participants described COPD-SG participation as fun because of the pleasurable sociable experience of singing together (McNaughton et al., 2016), because the groups provided exercise that was more interesting and welcoming than the gym (Clift et al., 2013; Lord et al., 2012) and because the atmosphere within the groups lacked the medicalised feel and connotations of pulmonary rehabilitation and patient support groups (McNaughton et al., 2016).

Alongside, within and beyond this fun and enjoyment, participants also described the process of singing together in COPD-SGs as affecting them in ways that transcended their usual emotional repertoires or experience, which they struggled to describe (Lewis and Thomas, 2018; McNaughton et al., 2016; Skingley et al., 2018). Studies struggled to conceptualise or to define this phenomenon, with Clift et al. (2013: 21) using the participant-reported terms ‘feel-good factor’, ‘adrenaline buzz’, ‘contribut[ion] to spiritual health’ and ‘feel[ing] uplifted’. Lord et al. (2012) sought to discover whether these benefits could be distinguished from those of other creative group activities by introducing to their study a control group who attended a film club rather than singing classes, and found that singing participants were significant more satisfied with the experience. However, their research does not directly address what appears to be the central role of exercise – and the exhilaration and empowerment which participants reported from managing the physical exertion of COPD-SGs sessions – within this phenomenon.

McNaughton et al. (2016) attribute this phenomenon (henceforth referred to as *uplifted-ness*) to the overcoming of social and cultural barriers which had enabled participants to transcend their lived experiences of illness and to sing together. They explain the potentially spiritual quality of this connectedness in terms of how singing in a group involves the coming together of different voices in harmony.

Lewis (Lewis and Thomas, 2018) offers a more physiological explanation for the phenomenon of *uplifted-ness*, arguing that participants are exhaling most effectively whilst singing, thereby suddenly receiving optimal capillary perfusion and brain activity. Nevertheless, Clift et al. (2013) and McNaughton et al. (2016) found that *uplifted-ness* altered participants’ ways of thinking about themselves and the world around them, in some cases providing participants with a new outlook on life (Skingley et al., 2013). Irrespective of its origins, *being uplifted* remains a central phenomenon.

### Being involved

Participants appeared to derive the most meaning, purpose, *togetherness* and *uplifted-ness* from COPD-SGs which offered them a degree of autonomy, self-determination or ownership over their group. Being involved appeared to intensify *togetherness* and *uplifted-ness* to a degree that could relieve some of the loneliness, isolation and social marginalisation and anxieties that many participants, like many people with COPD, reported (Williams and Carel, 2018).

Participants attending the SfBRG classes (Lewis and Thomas, 2018; Lord et al., 2012) were offered little user involvement in their COPD-SG, which was managed and delivered by the hospital physiotherapy service. The high proportion of eligible patients who declined to participate in the study (82%), together with the significant levels of reported dissatisfaction suggests that the study itself might have benefitted from more public and patient involvement (PPI).

Although the two SdHRC studies followed a similar model to one another, the COPD-SGs created for the first study ended following the final evaluation. Participant responses to this were mixed, with one reporting feeling abandoned and another asserting a determination to re-establish the group (Skingley et al., 2013). The SdHRC team then engaged with PPI feedback from this first study in embedding sustainability, participation and ownership within the design and aims of the second study (Clift et al., 2017). Participants reported more positive experiences, and in particular a greater degree of *togetherness* and *uplifted-ness*, within the COPD-SGs of this second study (Clift et al., 2017).

The study of McNaughton et al. (2016) sought to establish a COPD-SG which would continue beyond the duration of their research on a participant-led, self-sustaining basis. They found, however, that autonomy was not something the group wanted, because participants valued the care they received from facilitators and the lack of demands placed upon them. Given the energy which the group had already invested in *being together*, this unwillingness to assume a further degree of ownership surprised the researchers. Within this phenomenon of *being involved*, the optimal level of participation, control or ownership therefore varied.

The effectiveness of this *being involved* lay both in the extent to which it enhanced the *being together* and *being uplifted* of the group and the degree to which this involvement empowered individuals and groups to become more physically and socially active within the wider communities. *Being involved* became particularly apparent in the project evaluation of the second SdHRC study (Clift et al., 2017) and in the videographic footage of the volunteering awards (NZMOH, 2016) received by participants of the McNaughton et al. study. The highest levels of friendship and support occurred within these groups, it could be argued, because the ethos of *involvement* encouraged participants to look to one another rather than to their singing teachers for help. It was within these COPD-SGs, too, that participants found most frequently *being uplifted* (McNaughton et al., 2016; Skingley et al., 2018). Within the UK studies, *being involved* provided individuals with the confidence and self-efficacy to leave the house more, to seek new career directions and to take more exercise. Within the New Zealand based study (McNaughton et al., 2016), the SYLO choir developed links with a local school choir and other nearby community organisations. Whenever this *being involved* extended beyond the usual boundaries of a patient group – for example when participants assumed control of the organising or funding the group, and when participants initiated links with other community groups – participants reported feelings of empowerment (McNaughton et al., 2016; Skingley et al., 2018).

## Discussion

### Being together

COPD-SGs benefit the collective wellbeing and health of groups and communities because the shared physical experience of the social body facilitates a collective embodiment (Scheper-Hughes, 1994; Scheper-Hughes and Lock, 1987), which enables individuals to develop intensive relationships that are able to transcend the marginalisation and vulnerabilities imposed by outside processes. Raw (2013) attributes the intensity of these relationships to the anthropological concept of *communitas. Communitas*, as described by Turner (1969), is the phenomenon which occurs when people brought together by marginalisation or adversity experience a strong collective sense of connectedness, equality and purpose. Within her study, Raw (2013) identifies AiH-generated *communitas* as originating particularly from the artistic and musical rituals which groups enact. This observation may explain why, within the COPD-SGs studied in this meta-ethnography, performing and rehearsing for performances played such a central role in strengthening the cohesion and identity of the group. However, the phenomenon of *being together* discovered by this meta-ethnography extended beyond the times of AiH-generated *communitas* generated by the harmonising of individual voices. Particularly in the UK-based studies, COPD-SG group members surpassed cultural obligation and social expectations in the way that they supported one another even beyond the boundaries of the groups (Skingley et al., 2013, 2018). Turner (1969) describes *communitas* as a socially subversive and change-orientated process that challenges inequality. Whereas the AiH movement has a long tradition of advocacy and activism (Stacy et al., 2002), the *being together* identified in this meta-ethnography involved little social consciousness or desire for social change; instead, COPD-SG members sought simply to support one another.

To locate the phenomenon of *being together* as a *body-social* within the more rigorously-studied concept of *communitas* would therefore be an over-simplification that would risk overlooking the unique features of both. Nevertheless, the parallels identified between *being together* and *communitas* may provide some understanding of why, particularly for the most vulnerable people, social and community connectedness is of such positive benefit to wellbeing and health. People with COPD are known to be particularly prone to social isolation, marginalisation and many forms of anxiety (Williams and Carel, 2018). Within all forms of health research, the importance of supportive relationships is well-evidenced, with loneliness and social isolation emerging as a significant public health challenge in both policy and practice (Cheng et al., 2020). COPD-SGs benefit the wellbeing and health of individuals because they facilitate social connectedness and supportive relationships, as do all group-based AiH activities (Fletcher et al., 2019).

### Being uplifted

Turner (2012) has expanded the concept of *communitas* to explore the phenomenon of what Ehrenreich (2007) describes as *collective joy* as a tool for healing within creative ritual and movements of counter-cultural social resistance. Like Raw, Ehrenreich (2007) focuses upon socially subversive artistic genres – and, within the modality of singing, specifically rock music – and upon the power of this social subversion to engender a group experience of *collective joy*. From the work of Ehrenreich (2007) and Turner (2012), parallels emerge between the phenomena of *collective joy* and what Clift et al. (2011) conceptualise as the spiritual benefits of singing; both involve previously marginalised people coming together and making meaning together as a group, and both produce tangible improvements to the physical and emotional wellbeing and health of participants.

Beyond Clift et al. (2011), no research has identified MiH activities as involving any aspects of spirituality, perhaps because their quantitative or realist methods render this a difficult area to study. From the limited points of comparison available, it is not possible to establish whether *being uplifted* is phenomenologically analogous to the spiritual benefits described by Clift et al. (2011). Within the Global North, spirituality beyond religion is generally considered to be an individual resource, even when, as described by Clift et al. (2011), it is derived from a group experience. Within the *being uplifted* of this review, as within the *collective joy* of Ehrenreich (2007) and Turner (2012), participants described a collectively embodied *body-social* rather than an individual *body-subject* experience.

Whereas the phenomena both of *collective joy* and of *being uplifted* involved groups transcending isolation and marginalisation to come together, COPD-SG experienced *being uplifted* without engaging in the forms of advocacy or activism that characterise the histories of *collective joy* and the AIH movement. Within the existentialist paradigm from which phenomenological understandings of the different facets of bodily experience arose, discussions around the benefits of collectively and less goal-directedly *being uplifted* become problematic. This is because the individuality of the body-subject has traditionally been regarded as the foremost agent of human consciousness, emotion and meaning making (Sartre, 1943). From a more clinical perspective, however, both Merleau-Ponty’s collective embodiment of *intercorporeity* (Merleau-Ponty, 1945) and more context-specific collectively established understandings and meanings around the body and illness may be more beneficial to groups and individuals in the way that they promote emotional and physical resilience to challenges and change, thereby enhancing wellbeing and health (Antonovsky, 1996). From both perspectives, the existential questioning and meaning making encouraged by collectively *being uplifted* in COPD-SGs could be commended for encouraging individuals and groups to take greater ownership and control over their bodies and illnesses. *Being uplifted* therefore leads existentially and phenomenologically to *being involved*.

### Being involved

Patient *involvement* in the delivery and governance of health services is known to benefit individuals and communities whenever it produces a genuinely meaningful sense of empowerment, ownership or autonomy, usually leading to more relevant and effective forms of healthcare provision (Tritter and McCallum, 2006). Both Macnaughton et al. (2005) and Raw (2013) describe this participation as a central component of the heritage and history of the AiH movement. This meta-ethnography explains *being involved* as instrumental to facilitating the communal or collective body social processes of *being together* and *being uplifted*.

Over the past 20 years, discourses of *involvement*, empowerment and the active patient have become embedded throughout the fields of social care, and to an extent health. However, practitioners and academics disagree amongst themselves as to what *involvement* should be (Beresford, 2012) and within what parameters and to what extent patients should be delegated control of their treatment or care (Greene and Hibbard, 2012; Tritter and McCallum, 2006). Whereas this meta-ethnography identified *being involved* as of central significance to the qualitative effectiveness of COPD-SGs, it is therefore important not to conflate this phenomenon with the broader and more contentious descriptions of *involvement* or the ‘activated patient’ found in the literature of health policy and research (Beresford, 2012; Greene and Hibbard, 2012). Because COPD-SGs operate largely beyond the structures of mainstream health provision, it is likely that the phenomenon of *being involved* affords COPD-SG participants a greater degree (Yoeli et al., 2021) of decision-making, authority and control than some *involvement* offered within statutory services. COPD has long been recognised as most prevalent within the most deprived marginalised social groups and is known to be associated with anxiety, mobility restrictions, low self-confidence and isolation (Macnaughton, 2020; Williams and Carel, 2018), From this perspective, it appears that participants for whom *being involved* in a COPD-SG affords them opportunities to assert their interpersonal, administrative or leadership skills will feel particularly empowered by their involvement. Being and participating, through all aspects of the COPD-SG processed, transformed the embodied experience.

### The strengths and limitations of this study

By applying phenomenological methods exclusively to the qualitative data, this meta-ethnography has foregrounded the process-driven and collective benefits of COPD-SGs that literature reviews using more systematic methods and more quantitative datasets have struggled to identify.

In common with all previous reviews of the widest qualitative benefits to music and singing (Clark and Harding, 2012; Clements-Cortés, 2015; Clift et al., 2011; Daykin et al., 2018; Reagon et al., 2016), this meta-ethnography offers extremely positive findings. This meta-ethnography’s congruence in tone and optimism with previous reviews should attest to its rigour and credibility: aside from the criticisms of group structure and design (Clift et al., 2013; Lord et al., 2012), studies found COPD-SGs groups to be unremittingly and unreservedly positive about their collective experiences of singing together. However, from a purely intuitive perspective, it is difficult to credit the assertion that there were no negative experiences. Given that participants in at least one study (that of Clift et al., 2017; Skingley et al., 2018) were aware that the continuation of their group depended upon positive evaluation results, it is likely that they were affected by some degree of the ‘double hermeneutic’ of reporting what they believed researchers wanted them to hear. Clift et al. (2011, 2013, 2017) considered at length the typical characteristics of COPD-SG participants, noting that they tend to be drawn from existing patient support groups. The optimism of this meta-ethnography’s findings may therefore derive from the self-selecting nature of the group.

Phenomenological approaches to research are often criticised as overly subjective or as lacking in scientific reliability or rigour. Nevertheless, the philosophical advantages inherent within phenomenology are such that the findings of this meta-ethnography offer a more optimistic and arguably more valid reality than the quantitative data. Because of the added epistemological scope that the phenomenological standpoint of this meta-ethnography has contributed – in identifying, for example, the concept of *uplifted-ness* and the significance of *being uplifted* – this meta-ethnography has been able to identify some of the wellbeing and health benefits to COPD-SGs that quantitative methods have been unable to capture. Research from a more realist standpoint might provide more context, and might explain which patients are likely most to experience the *togetherness*, *uplifted-ness* and meaningful *involvement* this research describes, in what circumstances and how.

COPD-SGs are increasingly becoming a global movement, and we recognise that, in drawing only from studies based in the UK and New Zealand, this meta-ethnography does not reflect this. As a theoretical tradition, phenomenology originates within the Western philosophical assumptions and debates around the existence and nature of the Self and the individuality of the human body (Csordas, 1993; Merleau-Ponty, 1945). As a result, health research studies containing the depth of qualitative analysis required by our search protocol tend to originate primarily from Europe, Australasia, North America and the more Westernised areas of the Global South. The Uganda-based study of Downes et al. (2019), which would nevertheless have met the inclusion criteria for this meta-ethnography, was not published until after our research was completed. Downes et al. (2019) found that ‘*concepts within the [SfB assessment] questionnaires were not easily translated to Ugandan cultural understandings of health, disease and symptoms*’ (p. 224), and that the SfB model could not straightforwardly be transposed to a Ugandan context. When considered alongside the early anthropological antecedents to our conceptualisation of the body-social (Scheper-Hughes and Lock, 1987), this cultural incongruity is perhaps inevitable; societies’ collective understandings of the body are informed by the world they embody. We acknowledge, then, the culture-bound Anglocentric bias of our study, and the resultant lack of global generalisability of our findings. As COPD-SG research becomes increasingly global – something evident also from the less qualitative Chinese study of Liu et al. (2019) –, it is becoming increasingly important to understand the relationship between COPD-SGs, communities and health within cultures which attach different meanings to concepts around music, breathing, illness and togetherness.

### The body-social: *Being* as a communal or collective process

The phenomenological standpoint from which this meta-ethnography has developed concepts around the social and communal aspects of being and embodiment, has enabled it to focus upon the benefits of *being together*, *being uplifted* and *being involved*, which operate on a communal or collective basis, rather than the conventional measures of impact on wellbeing or health interventions upon individuals. COPD-SGs are at their most effective on a communal or collective level because they approach the body as a social as well as an individual entity, endorsing Heidegger’s foundational aim of identifying the gap between phenomena and reality. The centrality of these communal or collective processes strengthens our argument that the *body-social* should be afforded parity of consideration with the *body-subject* and *body-object* in phenomenological research that seeks to engage with group-based health interventions.

## Conclusion

Qualitative research into the wellbeing and health benefits of COPD-SGs offers more positive results because qualitative methods can discover what quantitative methods largely cannot. By identifying *being* as a collective phenomenon, and by identifying three phenomena of *being together, being uplifted* and *being involved*, this meta-ethnography offers findings distinct from previous reviews of the impact of COPD-SGs, which took the more empirically realist perspective of some of the organisations and services within which COPD-SGs operate. Not all COPD-SGs, and not all COPD-SG facilitators would identify themselves with the AiH movement or culture, sometimes preferring to describe themselves as healthcare interventions or social prescribing initiatives. Nevertheless, the AiH movement, from within which the concept of COPD-SGs and the mechanism of social prescribing originated, held a strongly collectively-focused ideology and ethos, seeking foremost to strengthen the wellbeing and health of communities by promoting collective identity, cohesion and consciousness as a means advocating social justice, involvement and activism. This meta-ethnography has demonstrated that, although the specific aims and techniques of COPD-SGs have developed ostensibly from a more individualised, behavioural or medicalised approach, and although COPD-SG members express no radical intent, the group-based heritage of the AiH movement remains nevertheless central to the power of COPD-SGs in improving wellbeing and health.

We argue, then, that this is a heritage and a tradition that COPD-SGs should embrace and draw from. COPD-SGs improve wellbeing largely through the cohesion and supportiveness of their groups. Those teaching, running or facilitating COPD-SG groups should consider how the structure of session and choice of activities might best support group members in bonding, socialising and developing a group identity together. COPD-SGs develop cohesion, supportiveness and identity most effectively when offered a meaningful level of ownership, self-determination or control over the delivery of the group. Those commissioning or managing COPD-SG services should consider a community choir model of service delivery to maximise their distinctiveness and value as groups, and to draw most fully from their heritage as facilitators of improved wellbeing. They will require some degree of autonomy from medical or rehabilitative services in order to delegate the necessary level of control to participants.

## Notes

1. from ‘You Raise Me Up’ (Graham, Løvland, and Evans, 2002): selected for performance by the *Singing for Better Breathing* choir, London UK, and by the *Sing Your Lungs Out* choir of Wellington, New Zealand, 2016.

2. McNaughton, the lead author of McNaughton et al. (2016), should not be confused with Macnaughton (Macnaughton and Carel, 2016; Macnaughton et al., 2005).
